# Why does type 2 diabetes mellitus impair weight reduction in patients with obesity? A review

**DOI:** 10.1016/j.obpill.2023.100076

**Published:** 2023-06-13

**Authors:** Harold Edward Bays

**Affiliations:** Diplomate of American Board of Medicine, Medical Director / President, Louisville Metabolic and Atherosclerosis Research Center, Clinical Associate Professor, University of Louisville School of Medicine, 3288 Illinois Avenue, Louisville, KY, 40213, USA

**Keywords:** Adiposopathy, Diabetes, Obesity, Weight reduction

## Abstract

**Background:**

A common adiposopathic complication of obesity is type 2 diabetes mellitus. Healthful weight reduction in patients with obesity can improve glucose metabolism and potentially promote remission of type 2 diabetes mellitus. However, weight-reduction in patients with increased adiposity is impaired among patients with type 2 diabetes mellitus compared to patients without diabetes mellitus.

**Methods:**

Data for this review were derived from PubMed and applicable websites.

**Results:**

Among patients with increased body fat, the mechanisms underlying impaired weight reduction for those with type 2 diabetes mellitus are multifactorial, and include energy conservation (i.e., improved glucose control and reduced glucosuria), hyperinsulinemia (commonly found in many patients with type 2 diabetes mellitus), potential use of obesogenic anti-diabetes medications, and contributions from multiple body systems. Other factors include increased age, sex, genetic/epigenetic predisposition, and obesogenic environments.

**Conclusions:**

Even though type 2 diabetes mellitus impairs weight reduction among patients with increased adiposity, clinically meaningful weight reduction improves glucose metabolism and can sometimes promote diabetes remission. An illustrative approach to mitigate impaired weight reduction due to type 2 diabetes mellitus is choosing anti-diabetes medications that increase insulin sensitivity and promote weight loss and deprioritize use of anti-diabetes medications that increase insulin exposure and promote weight gain.

## 1 Introduction

Type 2 diabetes impairs weight reduction among those with increased adiposity. The mechanisms accounting for this common clinical finding are multifactorial. Factors include energy conservation due to improvement in blood glucose control and reduced glucosuria, hyperinsulinemia commonly found in patients with type 2 diabetes mellitus, potential use of obesogenic anti-diabetes medications, and contributions from multiple body systems. Other factors include increased age, sex, genetic/epigenetic predisposition, and obesogenic environments. From a pharmacologic standpoint, the choice of anti-diabetes medications should prioritize those that increase insulin sensitivity and promote weight reduction and deprioritize anti-diabetes medications that increase insulin exposure and promote weight gain. Table 1 provides a summary of ten things to know about how type 2 diabetes mellitus impairs weight reduction among patients with increased adiposity.

**Table 1:** Ten things to know about how type 2 diabetes mellitus impairs weight reduction among patients with increased adiposity.

1. The presence of type 2 diabetes mellitus impairs weight reduction among patients with obesity treated with healthful nutrition, physical activity, behavior modification, and medical treatment (i.e., anti-obesity medications and bariatric surgery).
2. When weight reduction improves glucose control in patients with diabetes mellitus, then energy is conserved due to reduced glucosuria.
3. Especially early in the onset of type 2 diabetes mellitus, insulin resistance results in hyperinsulinemia. Insulin stimulates lipid uptake and storage in adipocytes and inhibits lipolysis, which are obesogenic mechanisms not found in patients who do not have insulin resistance.
4. Many patients with type 2 diabetes mellitus are treated with obesogenic medications (e.g., insulin, sulfonylureas, meglitinides, and thiazolidinediones). Additionally, administration of the first three listed hypoglycemic medications (i.e., anti-diabetes mellitus medications that increase insulin exposure) may contribute to hypoglycemia, necessitating increased caloric intake to avoid or treat low blood sugar. Increasing caloric consumption to treat or avoid hypoglycemia may impair weight reduction compared to patients without diabetes mellitus who do not require increased caloric consumption to treat or avoid hypoglycemia.
5. Body system abnormalities that may contribute to impaired weight reduction in patients with type 2 diabetes mellitus include endocrine, musculoskeletal, neurological, cardiovasculo-pulmonary, immune, ophthalmologic, nephrologic, dermatologic, and gastrointestinal.
6. Patients with type 2 diabetes mellitus are often older than patients without type 2 diabetes mellitus. Increasing age impairs weight reduction.
7. Patients with type 2 diabetes mellitus may have sex and genetic/epigenetic predisposition to obesity, which may impair weight reduction.
8. Obesogenic mechanisms that may contribute to impaired weight reduction among patients with type 2 diabetes mellitus include established meal planning, socioeconomic factors, psychosocial factors, bias, and discrimination.
9. Irrespective of the weight reduction challenges with the presence of type 2 diabetes mellitus, each of the four pillars of obesity management [healthful nutrition, physical activity, behavior modification, and medical therapy (i.e., anti-obesity medications and bariatric surgery)] can achieve clinically meaningful weight reduction among many patients with type 2 diabetes mellitus, potentially promoting remission of type 2 diabetes mellitus.
10. Pharmacologic treatment of type 2 diabetes mellitus should prioritize use of anti-diabetes medications that increase insulin sensitivity, reduce insulin levels, and reduce body weight (e.g., metformin, glucagon-like peptide-1 based therapies, sodium glucose- cotransporter 2 inhibitors), and deprioritize use of anti-diabetes medications that increase insulin exposure and increase body weight (e.g., sulfonylureas, insulin, meglitinides).

## 2 Obesity and adiposopathy as a cause of diabetes mellitus

**Fig. 1:**
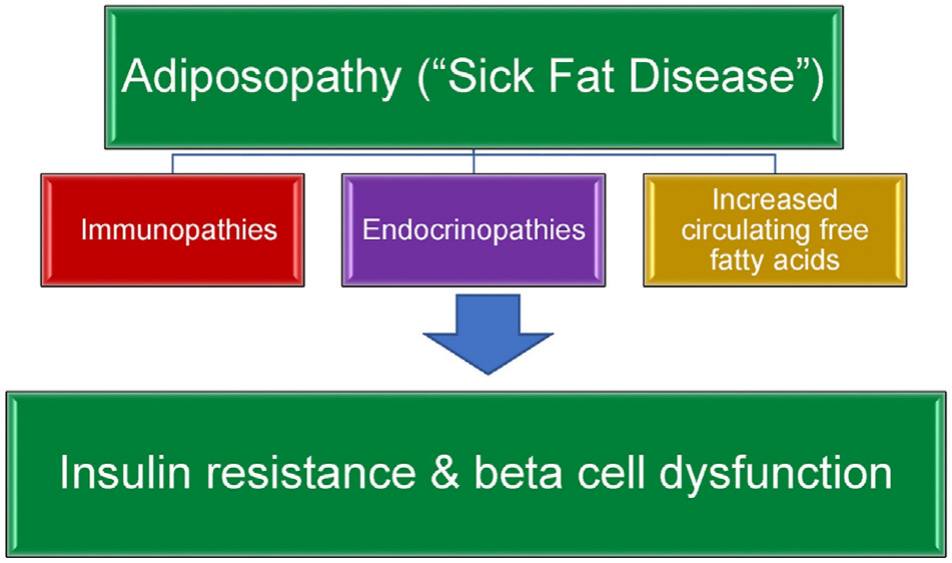
Ad**iposopathy contributes to type 2 diabetes mellitus.** See text for details (Copied with permission from Bays HE et al. Obesity Pillars https://doi.org/10.1016/j.obpill.2023.100056 [1]).

Details explaining the adiposopathic consequences of obesity that cause diabetes mellitus were previously described [1]. In summary, Fig. 1 describes how obesity may result in adiposopathy (“sick fat disease”), defined as “pathogenic adipose tissue anatomic/functional derangements, promoted by positive caloric balance in genetically and environmentally susceptible individuals, that result in adverse endocrine and immune responses that directly and/or indirectly contribute to metabolic diseases (e.g., type 2 diabetes mellitus, hypertension, dyslipidemia, cardiovascular disease, and cancer)” [2].

**Fig. 2:**
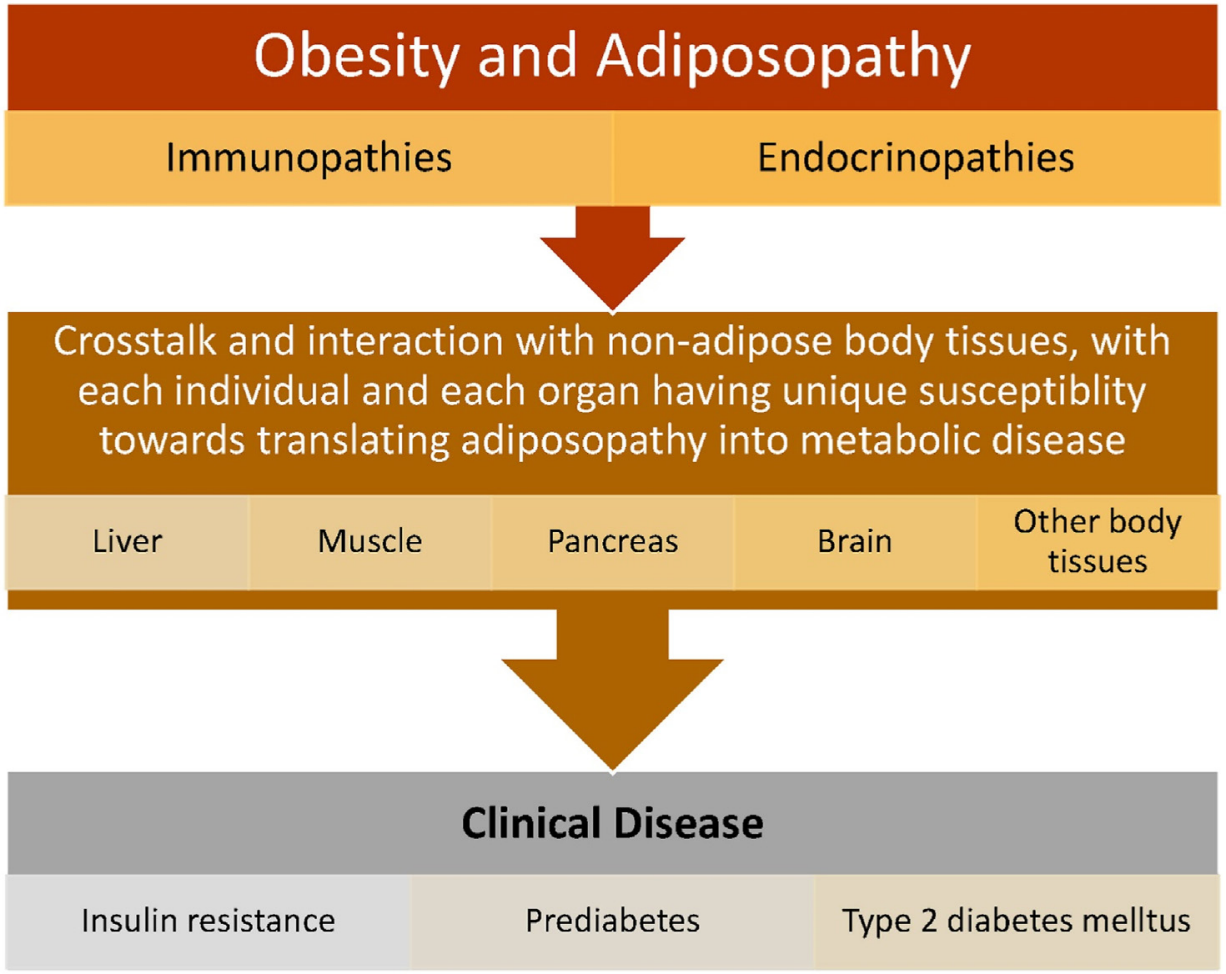
**Importance of non-adipose tissue in obesity-related glucose dysregulation and other cardiometabolic diseases.** See text for details. Copied with permission from Bays HE et al. Obesity Pillars https://doi.org/10.1016/j.obpill.2023.100056 [1]).

Fig. 2 describes the importance of adipose tissue crosstalk and biometabolic interactions with organs such as liver, muscle, pancreas, kidney, and brain. Adiposopathic immunopathies, endocrinopathies, and increased circulating free fatty acids contribute to multi-organ insulin resistance, as well as an ultimate decline in pancreatic beta cell insulin secretory function. Among patients with pre-obesity/overweight or obesity, the degree that weight reduction improves adverse clinical outcomes may vary, with improvement in glucose metabolism in some patients occurring with as little as ≥ 2% weight reduction. Greater amounts of weight reduction are generally required for reduction in cardiovascular disease and overall mortality [1,3].

**Fig. 3:**
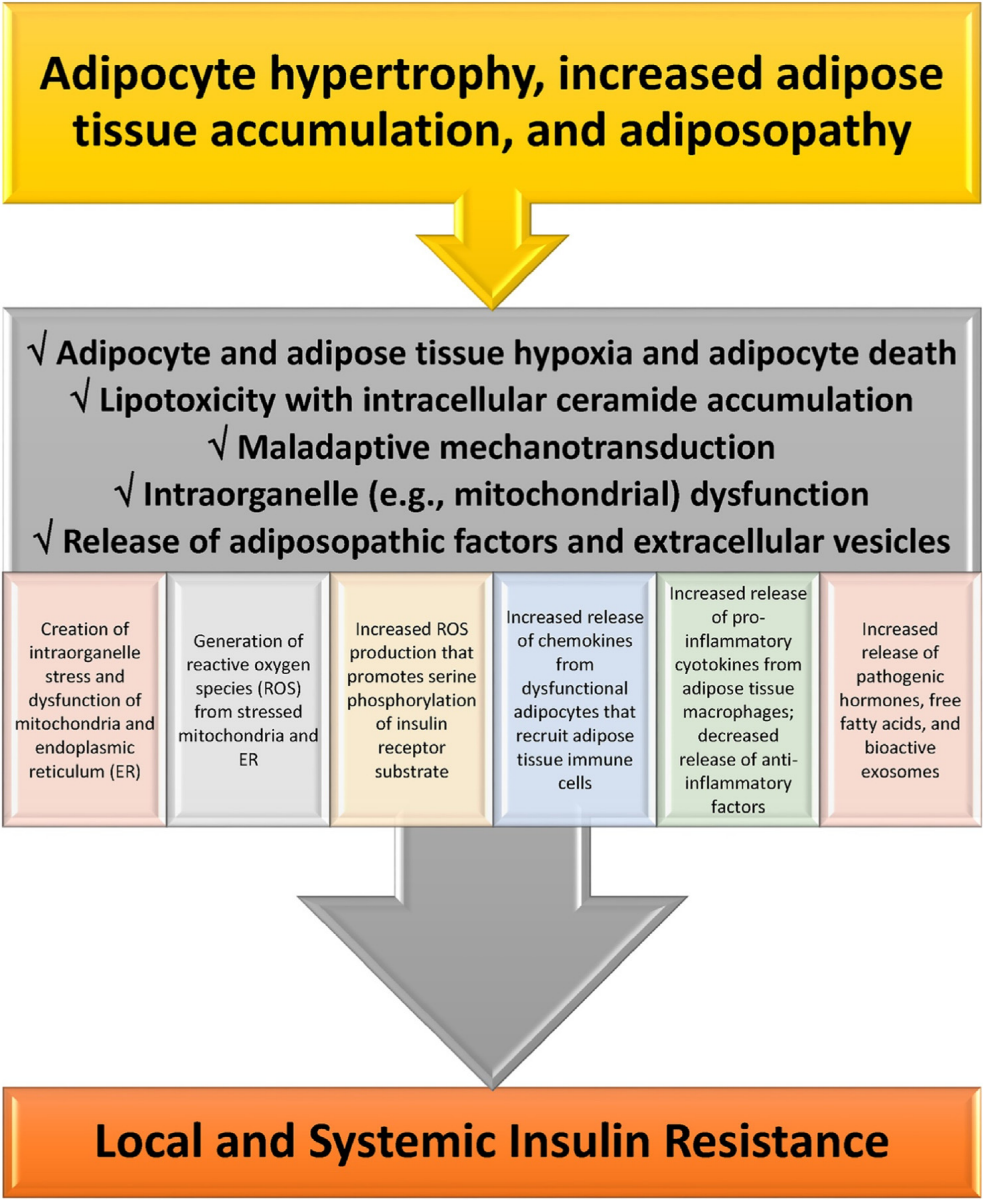
**Mechanisms how adiposopathic processes lead to insulin resistance.** See text for details (Copied with permission from Bays HE et al. Obesity Pillars https://doi.org/10.1016/j.obpill.2023.100056 [1]).

Fig. 3 describes that when positive caloric balance leads to pathogenic adipocyte hypertrophy, and when adipose tissue accumulation outgrows vascular supply, then this may result in adipocyte and adipose tissue hypoxia that potentially contributes to adipocyte death and adiposopathic effects on angiogenesis, adipocyte proliferation, adipocyte differentiation, reactive oxygen species generation, inflammation, and fibrosis. Excessive accumulation of intracellular lipids (i.e., ceramides diacylglycerol) may lead to lipotoxicity, adipocyte dysfunction, and insulin resistance. Another potential maladaptive process of adipose tissue dysfunction during positive caloric balance is disruption of mechanotransduction, which is the ongoing adaptation of adipose tissue to its microenvironment (e.g., formation, dissolution, and reformation of extracellular matrix). Continuous adipose tissue remodeling is required to maintain structural and functional integrity. If during positive caloric balance, adipose tissue undergoes fat cell hypertrophy, immune cells infiltration, fibrosis and changes in vascular architecture, impaired adipose tissue expansion, then this may limit further energy storage in adipose tissue, and thus promote additional adipose tissue dysfunction or adiposopathy.

Reactive oxygen species (ROS) are unstable oxygen-containing molecules that may damage deoxynucleic acid, adversely affect macromolecules, and disrupt cellular function. ROS contribute to insulin resistance, diabetes mellitus, cardiovascular diseases, atherosclerosis, cancer, and aging [1,4,5]. Along with hyperglycemia, adiposopathic processes that may generate ROS include mitochondrial and endoplasmic reticulum stress. Finally, beyond the circulatory release of adiposopathic proinflammatory factors, pathogenic hormones, and free fatty acids from adipose tissue, is the release of such factors from adipocyte extracellular vesicles.

Collectively, these adiposopathic processes [1] contribute to systemic insulin resistance in organs such as liver and muscle [6], with possible subsequent beta cell failure [7], and impaired weight reduction among patient with pre-obesity/overweight or obesity.

## 3 Obesity management and improvement in glycemic control, including remission of type 2 diabetes mellitus

Irrespective of impaired weight reduction with the presence of type 2 diabetes mellitus among patients with increased body fat, an essential clinical message is that body fat reduction may not only improve glucose metabolism, but obesity management may also result in diabetes remission [1].

• **Nutritional intervention:** Type 2 diabetes mellitus is most likely to undergo remission among patients who achieve clinically meaningful weight reduction, regardless of diet type [1].
• **Weight management program**: Type 2 diabetes mellitus remission is most likely to be maintained with sustained weight reduction [1].
• **Bariatric surgery**: Type 2 diabetes mellitus remission with bariatric surgery is most likely to occur with greater weight reduction [1].

Thus, regarding the clinical management of patients with increased body fat, impaired weight reduction among those with type 2 diabetes mellitus should not deter the recommendation and implementation of interventions to achieve a healthier body weight. Clinically meaningful weight reduction can help achieve improved fat mass disease and improved sick fat disease, thus improving glucose control and other cardiometabolic parameters [2].

## 4 Weight reduction among patients with pre-obesity/overweight and/or obesity, with and without diabetes mellitus

Not all weight reduction interventions in patients with increased adiposity have head-to-head comparisons of efficacy among those with type 2 diabetes mellitus, versus those without type 2 diabetes mellitus. However, a general overview of the data suggests that weight reduction interventions are less effective among those with type 2 diabetes mellitus than without type 2 diabetes mellitus [8]. While the quality of the data, as well as statistic and clinical significance vary, the amount of weight reduction and/or success of weight reduction maintenance appears less among those with type 2 diabetes mellitus treated with dietary intervention, physical activity, behavior modification [9,10],orlistat [11], phentermine [12], phentermine/topiramate [13,14], naltrexone/bupropion [13,[15], [16], [17]], liraglutide [13,18], semaglutide [19], as well as bariatric surgery [20]. It may also be relevant that in the tirzepatide SURMOUNT program [21], SURMOUNT 1 demonstrated weight reduction up to 21% among patients with overweight/obesity and without diabetes mellitus [22], while preliminary reports suggest that in SURMOUNT 2, tirzepatide reduced weight up to 16% among patients with overweight/obesity and type 2 diabetes mellitus (https://investor.lilly.com/node/48776/pdf).

## 5 Potential mechanisms for impaired weight reduction among patients with increased adiposity and type 2 diabetes mellitus

### 5.1 Clinical mechanisms (See Fig. 4)

Improvement in glucose blood levels may impair weight reduction through improved energy conservation and decreased glucosuria. In patients without diabetes mellitus, filtered glucose is typically reabsorbed in renal tubules such that glucosuria only occurs when blood sugars exceed a variable threshold of around 180 mg/dL [23]. When blood sugars exceed this renal threshold, then this results in a loss of calories due to an increase in urine glucose excretion (4 kcal/g of glucose) [24]. “Normal” urine glucose excretion is less than 25 mg/dL [25]. In patients with poorly controlled diabetes mellitus, high blood sugars can result in spot urine glucose levels as high as ≥ 1000 mg/dL. Additionally, poorly controlled diabetes mellitus not only results in caloric/energy loss via glucosuria, osmotic water weight loss due to glucosuria, but also weight loss due to loss of water-associated glycogen in muscle [3]. Among patients with diabetes mellitus, especially those with poorly controlled blood sugars, anti-obesity interventions that improve glucose metabolism will not only conserve energy by reducing glucosuria, but also reverse the osmotic dehydration, replenish muscle glycogen, and increase its associated water, which all contribute to weight gain. While beneficial from a health perspective, each of these treatment effects that reduce hyperglycemia mitigate weight reduction.

Another relevant clinical mechanism impairing weight reduction is the use of obesogenic anti-diabetes medications such as insulin, sulfonylureas, meglitinides, and thiazolidinediones [26,27]. Thiazolidinediones are peroxisome proliferator gamma agonists agents that as part of their mechanism of action, reduce glucose levels through increasing the proliferation and differentiation of adipocytes, adding functional adipocytes, and thus mitigating adiposopathy [28]. While the increase in body weight with thiazolidinediones in partially fluid weight gain, much of the weight gain is due to increased (functional) body fat [28,29]. Insulin, sulfonylureas, and meglitinides are hypoglycemic therapies that increase insulin exposure. It is not uncommon that patients treated with insulin can gain 7–20 pounds the first year after initiating insulin therapy [30]. Furthermore, the use of anti-diabetes medications that increase insulin exposure often promote hypoglycemia (particularly during times of weight reduction), requiring increased caloric consumption to treat low blood sugars. Increased caloric consumption to avoid or treat hypoglycemia counteracts the effectiveness weight reduction efforts. In fact, hypoglycemia is predictive of weight gain with insulin therapies, although weight gain and hypoglycemia may be less with some insulin formulations, such as insulin detemir [31].

As noted in Fig. 1, Fig. 2, Fig. 3, insulin resistance is a sentinel pathogenic mechanism, especially in early onset type 2 diabetes mellitus. Prior to the beta cell failure that often occurs over time, pancreatic beta cells respond to insulin resistance with increased insulin secretion, resulting in hyperinsulinemia. Insulin stimulates lipid uptake and storage and inhibits lipid breakdown [32]. Thus, the hyperinsulinemia found early in type 2 diabetes mellitus would seem to be a straight-forward mechanism driving or maintaining fat weight gain among patients with type 2 diabetes mellitus, compared to patients without hyperinsulinemia. However, the relationship between hyperinsulinemia and obesity is complex [32]. For example, if body tissues (including adipocytes) are “resistant” to the effects of insulin, then even if insulin blood levels were elevated, how would an increase in insulin levels still drive fat accumulation? One explanation is that hyperinsulinemia is the result of total body insulin resistance that occurs not only from insulin resistance in adipose tissue, but also from insulin resistance in other body organs such as muscle and liver. Prior to onset of type 2 diabetes mellitus, skeletal muscle appears to be more sensitive to insulin than the liver and adipose tissue [33]. Skeletal muscle is the major organ responsible for post-meal glucose disposal [34]. Conversely, early development of type 2 diabetes mellitus, skeletal muscle insulin resistance is a defect that may occur before insulin resistance in the liver and adipose tissue [33]. Especially when the insulin resistance in muscle exceeds that of adipose tissue, then adipocyte responsiveness to hyperinsulinemia may be maintained in a relative sense, with the potential to promote lipogenesis, suppress lipolysis, and increase lipid storage in adipocytes [35]. These effects of hyperinsulinemia on promoting fat deposition might reasonably be concluded to counteract body fat reduction efforts.

**Fig. 4:**
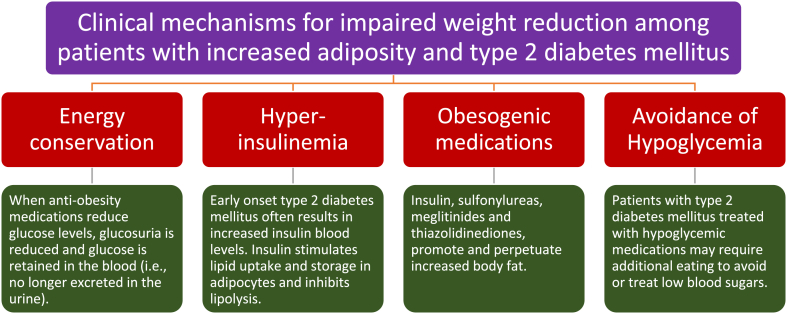
**Proposed clinical mechanisms to explain impairment of weight reduction among patients with increased adiposity and type 2 diabetes mellitus.** (See text for details).

**Fig. 5:**
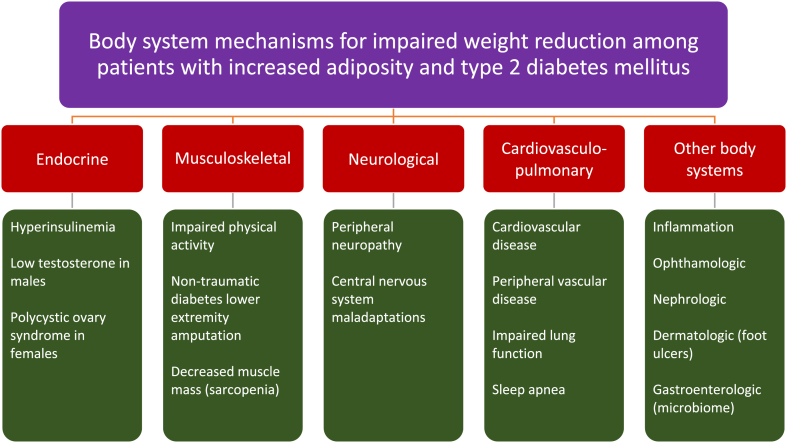
**Proposed body system mechanisms to explain impairment of weight reduction among patients with increased adiposity and type 2 diabetes mellitus**. (See text for details).

**Fig. 6:**
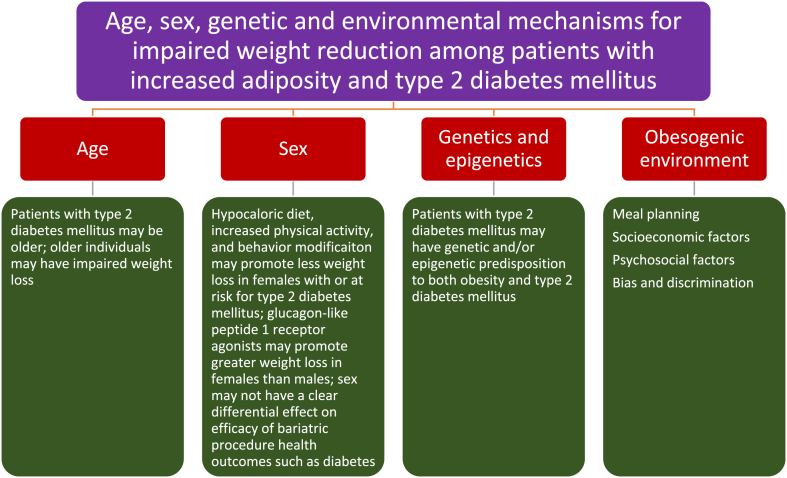
**Proposed age, sex, genetic/epigenetic, and environmental mechanisms to explain impairment of weight reduction among patients with increased adiposity and type 2 diabetes mellitus.** (See text for details).

### 5.2 Body system mechanisms (See Fig. 5)

From an endocrine standpoint, hyperinsulinemia may drive fat deposition as discussed in the previous section. Another potential endocrine abnormality that may occurs in males with type 2 diabetes mellitus is a reduction in testosterone levels. Overall, patients with type 2 diabetes have lower testosterone levels than patients without type 2 diabetes mellitus [[36], [37], [38]]. Obesity and type 2 diabetes may contribute to low testosterone, and low testosterone may contribute to increased body fat and decreased muscle mass [37] – both effects that may counter efforts to achieve a healthy body composition [39]. Females with polycystic ovary syndrome (PCOS) have an increased risk of type 2 diabetes [40], with PCOS being both a potential consequence (i.e., due to adiposopathic endocrinopathies) [2], and potential contributor to fat weight gain (i.e., via insulin resistance and associated eating disorders, depression, and anxiety) [[41], [42], [43], [44], [45], [46]].

Other organ systems disrupted by type 2 diabetes mellitus that may contribute impaired weight reduction among patients with increased adiposity include.

• Diabetes-related musculoskeletal abnormalities that may impair physical activity include fibroproliferative disorders of soft tissue, joint disorders, muscle-related disorders, and skeletal disorders [47]. Type 2 diabetes mellitus lower extremity amputation may also limit physical activity, and may be associated with a high prevenance of obesity [48,49].
• Diabetes-related peripheral neuropathy may impair physical activity and increase visceral fat accumulation [50]. Diabetes-related central nervous system abnormalities may potentially increase hunger [51]. While longstanding dementia may be associated with weight loss, obesity, diabetes mellitus, and dementia have overlapping etiologies [52], suggesting the need for early healthful nutrition to simultaneously address all three through evidenced-based medical nutrition therapies such as Mediterranean Diet, Dietary Approaches to Stop Hypertension (DASH) Diet, or Mediterranean-DASH Intervention for Neurodegenerative Delay (MIND) diet [[53], [54], [55]].
• Diabetes-related cardiovasculo-pulmonary abnormalities may impair physical activity due to the spectrum of diabetes heart diseases [56,57], stroke [58], and peripheral vascular disease [58]. Diabetes mellitus is also associated with impaired lung function [59], which may impair physical activity. More than ½ of patients with type 2 diabetes mellitus having obstructive sleep apnea [60], with sleep apnea contributing to weight gain and visceral fat accumulation [[61], [62], [63]].
• Diabetes-related inflammation are interrelated with obesity [64], with diabetes being potentially associated with hypothalamic inflammation [65], which in turn is also linked to promoting obesity [66,67].
• Diabetes-related eye disease [68], kidney disease [69], and skin disease (i.e., diabetes foot ulcer) [70] may impair physical activity, thus potentially impair weight reduction effectiveness.
• Diabetes-related abnormalities in the microbiome may contribute to insulin resistance [71], potentially impairing weight reduction.

### 5.3 Age, sex, genetic/epigenetic, and environmental mechanisms (See Fig. 6)

• Compared to those without diabetes, some data suggests that type 2 diabetes mellitus may modestly increase resting metabolic rate, but decrease insulin-induced thermogenesis [72,73]. That said, resting metabolic rate decreases with increasing age [74]. Physical activity also decreases with increasing age [75]. Thus older age is often associated with pathogenic alterations in body composition such as an increase in body fat and decrease in muscle mass [76,77], which are directional affects that would not favor fat weight reduction. Incident diabetes (and other chronic diseases) are associated with a steeper decline in resting metabolic rate over time [78]. Finally, the prevalence of prediabetes and type 2 diabetes mellitus are higher in older individuals [79]. Thus, an additionally clinically relevant mechanism why a patient with type 2 diabetes mellitus may have impaired fat weight loss could be because of older age.
• Insufficient data exists to make definitive statements regarding the effects of sex (i.e., female versus male) on the potential for weight reduction among patients with increased body fat and type 2 diabetes mellitus. However, males with prediabetes may lose more weight with low energy diets than females [80]. In the Diabetes Prevention Program, intensive lifestyle modification (i.e., at least 7% of weight loss through dietary modification and 150 min/week of moderate intensity exercise) resulted in greater weight loss in males versus females [81]. Males with type 2 diabetes mellitus may lose more weight than females with behavior modification [82]. Regarding glucagon-like peptide 1 receptor agonists, females may lose more weight than males [83]. Finally, regarding bariatric procedures, sex may not have a clear differential effect on efficacy outcomes, such as amount of weight reduction and resolution of obesity complications such as diabetes, hypertension, or sleep apnea [84].
• Genetic abnormalities [3,85] and disruption of epigenetic processes such as deoxynucleic acid methylation, histone modification, and ribonucleic acid processes may contribute to both obesity and type 2 diabetes mellitus [86,87]. Those with a genetic/epigenetic predisposition to both obesity and type 2 diabetes mellitus might conceivably experience greater challenges in achieving weight reduction compared to individuals without such genetic/epigenetic predisposition.
• Environments that promote obesity are often similar to environments that predispose to type 2 diabetes mellitus. Such environments are often difficult to resolve, and thus may impair the effectiveness of weight reduction interventions. One practical eating environment is the setting where the patient with type 2 diabetes has undergone years of reinforced education to consume three meals a day and a nighttime snack. It may be true that time-restricted eating may not achieve greater reduction in body weight, body fat, or metabolic risk factors compared to comparable daily calorie restriction [88]. However, if time restricted eating aids in reducing total daily caloric intake, then this may help achieve weight reduction [89]. Compared to the patient without diabetes mellitus, time restricted eating may more challenging for patients with type 2 diabetes mellitus who have undergone years of education, and years of acclimation regarding consuming three meals a day and nighttime snack. Other challenging promoters of both obesity and type 2 diabetes mellitus include socioeconomic status [90], psychosocial factors [91], as well as bias, and discrimination [92,93].

## 6 Conclusion

Obesity and type 2 diabetes mellitus are diseases that are often mechanistically interrelated [1], making it sometimes challenging to determine the degree that each independently contributes to adverse clinical consequences. Data is often lacking to definitively conclude how and why patients with type 2 diabetes mellitus and obesity seem to have greater difficulty with weight reduction than those with obesity without type 2 diabetes mellitus. Potential explanations described herein include clinical mechanisms, body system mechanisms, as well as age, sex, genetic/epigenetic, and environmental mechanisms. It may be diagnostically useful to know the potential mechanisms that might impair weight reduction among patients with type 2 diabetes mellitus and pre-obesity/overweight and obesity. However, what may be most clinically relevant is that clinically meaningful weight reduction can be achieved in patients with increased adiposity and type 2 diabetes mellitus via healthful nutrition, physical activity, behavior modification and medical therapy (i.e., anti-obesity medications and bariatric surgery). In some cases, these interventions have the potential to promote diabetes remission. Among patients with increased body fat, impaired weight reduction due to the presence of type 2 diabetes mellitus may be mitigated by choosing anti-diabetes medications that increase insulin sensitivity and promote weight loss and deprioritize use of anti-diabetes medications that increase insulin exposure and promote weight gain. This approach is a practical, illustrative example of how to apply the knowledge of why type 2 diabetes mellitus may impair weight reduction among patients with concurrent pre-obesity/overweight and/or obesity.

## Ethics review

This submission did not involve human test subjects or volunteers. Harold Bays MD (Editor-in-Chief) had no involvement in the peer-review and acceptance/rejection of this submission. Responsibility for the editorial process for this article was delegated to a non-author Editor.

## Author contributions

HEB created and edited this manuscript based upon peer review comments.

## Individual disclosures

HEB's research site/institution has received research grants from 89Bio, Allergan, Alon Medtech/Epitomee, Altimmune, Amgen, Anji Pharma, AstraZeneca, Bionime, Boehringer Ingelheim, Eli Lilly, Esperion, Evidera, GlaxoSmithKline, HighTide, Home Access, Ionis, Kallyope, LG-Chem, Madrigal, Merck, New Amsterdam, Novartis, NovoNordisk, Pfizer, Satsuma, Selecta, Shionogi, TIMI, and Vivus. HEB has served as a consultant/advisor for 89Bio, Altimmune, Amgen, Boehringer Ingelheim, HighTide, Lilly, and Esperion, and speaker for Esperion.

## Funding and acknowledgements

This manuscript received no funding.

## Declaration of AI and AI-assisted technologies in the writing process

During the preparation of this work the author(s) used Chat GPT to help list and categorize content. After using this tool/service, the author(s) reviewed and edited the content as needed and take(s) full responsibility for the content of the publication.

## References

[1] Bays H.E., Bindlish S., Clayton T.L. (2023). Obesity, diabetes mellitus, and cardiometabolic risk: an obesity medicine association (OMA) clinical practice statement (CPS) 2023. Obesity Pillars.

[2] Fitch A.K., Bays H.E. (2022). Obesity definition, diagnosis, bias, standard operating procedures (SOPs), and telehealth: an Obesity Medicine Association (OMA) Clinical Practice Statement (CPS) 2022. Obesity Pillars.

[3] Bays H.E., Golden A., Tondt J. (2022). Thirty obesity myths, misunderstandings, and/or oversimplifications: an obesity medicine association (OMA) clinical practice statement (CPS) 2022. Obesity Pillars.

[4] Alfadda A.A., Sallam R.M. (2012). Reactive oxygen species in health and disease. J Biomed Biotechnol.

[5] Lazarus E., Bays H.E. (2022). Cancer and obesity: an obesity medicine association (OMA) clinical practice statement (CPS) 2022. Obesity Pillars.

[6] Pearson T., Wattis J.A., King J.R., MacDonald I.A., Mazzatti D.J. (2016). The effects of insulin resistance on individual tissues: an application of a mathematical model of metabolism in humans. Bull Math Biol.

[7] Cerf M.E. (2013). Beta cell dysfunction and insulin resistance. Front Endocrinol.

[8] Franz M.J. (2017). Weight management: obesity to diabetes. Diabetes Spectr.

[9] Guare J.C., Wing R.R., Grant A. (1995). Comparison of obese NIDDM and nondiabetic women: short- and long-term weight loss. Obes Res.

[10] Wing R.R., Marcus M.D., Epstein L.H., Salata R. (1987). Type II diabetic subjects lose less weight than their overweight nondiabetic spouses. Diabetes Care.

[11] Tong P.C., Lee Z.S., Sea M.M., Chow C.C., Ko G.T., Chan W.B. (2002). The effect of orlistat-induced weight loss, without concomitant hypocaloric diet, on cardiovascular risk factors and insulin sensitivity in young obese Chinese subjects with or without type 2 diabetes. Arch Intern Med.

[12] Ashy A. (2017). The effect of phentermine on weight loss in diabetic patients. Boston University Theses & Dissertations.

[13] Coelho C., Agius R., Crane J., McGowan B. (2021). Pharmacotherapy for weight loss in adults with type 2 diabetes: a systematic review of randomised controlled trials. Br J Dermatol.

[14] Garvey W.T., Ryan D.H., Bohannon N.J., Kushner R.F., Rueger M., Dvorak R.V. (2014). Weight-loss therapy in type 2 diabetes: effects of phentermine and topiramate extended release. Diabetes Care.

[15] Greenway F.L., Fujioka K., Plodkowski R.A., Mudaliar S., Guttadauria M., Erickson J. (2010). Effect of naltrexone plus bupropion on weight loss in overweight and obese adults (COR-I): a multicentre, randomised, double-blind, placebo-controlled, phase 3 trial. Lancet.

[16] Apovian C.M., Aronne L., Rubino D., Still C., Wyatt H., Burns C. (2013). A randomized, phase 3 trial of naltrexone SR/bupropion SR on weight and obesity-related risk factors (COR-II). Obesity.

[17] Hollander P., Gupta A.K., Plodkowski R., Greenway F., Bays H., Burns C. (2013). Effects of naltrexone sustained-release/bupropion sustained-release combination therapy on body weight and glycemic parameters in overweight and obese patients with type 2 diabetes. Diabetes Care.

[18] Pi-Sunyer X., Astrup A., Fujioka K., Greenway F., Halpern A., Krempf M. (2015). A randomized, controlled trial of 3.0 mg of liraglutide in weight management. N Engl J Med.

[19] Jensterle M., Rizzo M., Haluzík M., Janež A. (2022). Efficacy of GLP-1 ra approved for weight management in patients with or without diabetes: a narrative review. Adv Ther.

[20] Adams S.T., Salhab M., Hussain Z.I., Miller G.V., Leveson S.H. (2013). Roux-en-Y gastric bypass for morbid obesity: what are the preoperative predictors of weight loss?. Postgrad Med.

[21] Bays H.E., Fitch A., Christensen S., Burridge K., Tondt J. (2022). Anti-obesity medications and investigational agents: an obesity medicine association (OMA) clinical practice statement (CPS) 2022. Obesity Pillars.

[22] Jastreboff A.M., Aronne L.J., Ahmad N.N., Wharton S., Connery L., Alves B. (2022). Tirzepatide once weekly for the treatment of obesity. N Engl J Med.

[23] Bays H. (2009). From victim to ally: the kidney as an emerging target for the treatment of diabetes mellitus. Curr Med Res Opin.

[24] Bays H.E., Weinstein R., Law G., Canovatchel W. (2014). Canagliflozin: effects in overweight and obese subjects without diabetes mellitus. Obesity.

[25] Cowart S.L., Stachura M.E., Walker H.K., Hall W.D., Hurst J.W. (1990). Clinical methods: the history, physical, and laboratory examinations.

[26] Tondt J., Bays H.E. (2022). Concomitant medications, functional foods, and supplements: an obesity medicine association (OMA) clinical practice statement (CPS) 2022. Obesity Pillars.

[27] Ghusn W., Hurtado M.D., Acosta A. (2022). Weight-centric treatment of type 2 diabetes mellitus. Obesity Pillars.

[28] Bays H.E. (2012). Adiposopathy, diabetes mellitus, and primary prevention of atherosclerotic coronary artery disease: treating "sick fat" through improving fat function with antidiabetes therapies. Am J Cardiol.

[29] Kushner R.F., Sujak M. (2009). Prevention of weight gain in adult patients with type 2 diabetes treated with pioglitazone. Obesity.

[30] Brown A., Guess N., Dornhorst A., Taheri S., Frost G. (2017). Insulin-associated weight gain in obese type 2 diabetes mellitus patients: what can be done?. Diabetes Obes Metabol.

[31] Davies M.J., Derezinski T., Pedersen C.B., Clauson P. (2008). Reduced weight gain with insulin detemir compared to NPH insulin is not explained by a reduction in hypoglycemia. Diabetes Technol Therapeut.

[32] Templeman N.M., Skovsø S., Page M.M., Lim G.E., Johnson J.D. (2017). A causal role for hyperinsulinemia in obesity. J Endocrinol.

[33] Mu W., Cheng X-f, Liu Y., Lv Q-z, Liu G-l, Zhang J-g (2019). Potential nexus of non-alcoholic fatty liver disease and type 2 diabetes mellitus: insulin resistance between hepatic and peripheral tissues. Front Pharmacol.

[34] Fujimoto B.A., Young M., Nakamura N., Ha H., Carter L., Pitts M.W. (2021). Disrupted glucose homeostasis and skeletal-muscle-specific glucose uptake in an exocyst knockout mouse model. J Biol Chem.

[35] Kolb H., Stumvoll M., Kramer W., Kempf K., Martin S. (2018). Insulin translates unfavourable lifestyle into obesity. BMC Med.

[36] Gianatti E.J., Grossmann M. (2020). Testosterone deficiency in men with Type 2 diabetes: pathophysiology and treatment. Diabet Med.

[37] Grossmann M. (2011). Low testosterone in men with type 2 diabetes: significance and treatment. J Clin Endocrinol Metabol.

[38] Kumari N., Khan A., Shaikh U., Lobes K., Kumar D., Suman F. (2021). Comparison of testosterone levels in patients with and without type 2 diabetes. Cureus.

[39] Burridge K., Christensen S.M., Golden A., Ingersoll A.B., Tondt J., Bays H.E. (2022). Obesity history, physical exam, laboratory, body composition, and energy expenditure: an Obesity Medicine Association (OMA) Clinical Practice Statement (CPS) 2022. Obesity Pillars.

[40] Wekker V., van Dammen L., Koning A., Heida K.Y., Painter R.C., Limpens J. (2020). Long-term cardiometabolic disease risk in women with PCOS: a systematic review and meta-analysis. Hum Reprod Update.

[41] Xu Y., Qiao J. (2022). Association of insulin resistance and elevated androgen levels with polycystic ovarian syndrome (PCOS): a review of literature. J Healthc Eng.

[42] Steegers-Theunissen R.P.M., Wiegel R.E., Jansen P.W., Laven J.S.E., Sinclair K.D. (2020). Polycystic ovary syndrome: a brain disorder characterized by eating problems originating during puberty and adolescence. Int J Mol Sci.

[43] Kolhe J.V., Chhipa A.S., Butani S., Chavda V., Patel S.S. (2022). PCOS and depression: common links and potential targets. Reprod Sci.

[44] Cooney L.G., Dokras A. (2017). Depression and anxiety in polycystic ovary syndrome: etiology and treatment. Curr Psychiatr Rep.

[45] Barber T.M., Hanson P., Weickert M.O., Franks S. (2019). Obesity and polycystic ovary syndrome: implications for pathogenesis and novel management strategies. Clin Med Insights Reprod Health.

[46] Christensen S.M., Varney C., Gupta V., Wenz L., Bays H.E. (2022). Stress, psychiatric disease, and obesity: an obesity medicine association (OMA) clinical practice statement (CPS) 2022. Obesity Pillars.

[47] Sözen T., Başaran N., Tınazlı M., Özışık L. (2018). Musculoskeletal problems in diabetes mellitus. Eur J Rheumatol.

[48] Littman A.J., McFarland L.V., Thompson M.L., Bouldin E.D., Arterburn D.E., Majerczyk B.R. (2015). Weight loss intention, dietary behaviors, and barriers to dietary change in veterans with lower extremity amputations. Disabil Health J.

[49] Mollee T.S., Dijkstra P.U., Dekker R., Geertzen J.H.B. (2021). The association between body mass index and skin problems in persons with a lower limb amputation: an observational study. BMC Muscoskel Disord.

[50] Oh T.J., Lee J.E., Choi S.H., Jang H.C. (2019). Association between body fat and diabetic peripheral neuropathy in middle-aged adults with type 2 diabetes mellitus: a preliminary report. J Obes Metab Syndr.

[51] Cugini P., Fatati G., Paggi A., Coaccioli S., Paci F., Palazzi M. (1996). Hunger sensation in patients with compensated and uncompensated type 1 and type 2 Diabetes Mellitus. Int J Eat Disord.

[52] Selman A., Burns S., Reddy A.P., Culberson J., Reddy P.H. (2022). The role of obesity and diabetes in dementia. Int J Mol Sci.

[53] Śliwińska S., Jeziorek M. (2021). The role of nutrition in Alzheimer's disease. Rocz Panstw Zakl Hig.

[54] Alexander L., Christensen S.M., Richardson L., Ingersoll A.B., Burridge K., Golden A. (2022). Nutrition and physical activity: an obesity medicine association (OMA) clinical practice statement 2022. Obesity Pillars.

[55] Liu X., Morris M.C., Dhana K., Ventrelle J., Johnson K., Bishop L. (2021). Mediterranean-DASH Intervention for Neurodegenerative Delay (MIND) study: rationale, design and baseline characteristics of a randomized control trial of the MIND diet on cognitive decline. Contemp Clin Trials.

[56] Ritchie R.H., Abel E.D. (2020). Basic mechanisms of diabetic heart disease. Circ Res.

[57] Crisafulli A., Pagliaro P., Roberto S., Cugusi L., Mercuro G., Lazou A. (2020). Diabetic cardiomyopathy and ischemic heart disease: prevention and therapy by exercise and conditioning. Int J Mol Sci.

[58] Mukherjee D. (2009). Peripheral and cerebrovascular atherosclerotic disease in diabetes mellitus. Best Pract Res Clin Endocrinol Metabol.

[59] Klein O.L., Krishnan J.A., Glick S., Smith L.J. (2010). Systematic review of the association between lung function and Type 2 diabetes mellitus. Diabet Med : a journal of the British Diabetic Association.

[60] Muraki I., Wada H., Tanigawa T. (2018). Sleep apnea and type 2 diabetes. J Diabetes Investig.

[61] Depner C.M., Stothard E.R., Wright K.P. (2014). Metabolic consequences of sleep and circadian disorders. Curr Diabetes Rep.

[62] Pennings N., Golden L., Yashi K., Tondt J., Bays H.E. (2022). Sleep-disordered breathing, sleep apnea, and other obesity-related sleep disorders: an Obesity Medicine Association (OMA) Clinical Practice Statement (CPS) 2022. Obesity Pillars.

[63] Bays H.E. (2022 Apr 5).

[64] Rohm T.V., Meier D.T., Olefsky J.M., Donath M.Y. (2022). Inflammation in obesity, diabetes, and related disorders. Immunity.

[65] Dong G.Z., Zhang Q.Y., Jiao Y.W., Ma Y., Zhu S.M., Zhang L.H. (2022). The contribution of type 2 diabetes mellitus to hypothalamic inflammation and depressive disorders in young patients with obesity. Ann Transl Med.

[66] Jais A., Brüning J.C. (2017). Hypothalamic inflammation in obesity and metabolic disease. J Clin Investig.

[67] Rahman M.H., Bhusal A., Lee W.H., Lee I.K., Suk K. (2018). Hypothalamic inflammation and malfunctioning glia in the pathophysiology of obesity and diabetes: translational significance. Biochem Pharmacol.

[68] Ong S.R., Crowston J.G., Loprinzi P.D., Ramulu P.Y. (2018). Physical activity, visual impairment, and eye disease. Eye.

[69] Wilkinson T.J., Clarke A.L., Nixon D.G.D., Hull K.L., Song Y., Burton J.O. (2021).

[70] Crews R.T., Schneider K.L., Yalla S.V., Reeves N.D., Vileikyte L. (2016). Physiological and psychological challenges of increasing physical activity and exercise in patients at risk of diabetic foot ulcers: a critical review. Diabetes Metab Res Rev.

[71] Chen X., Devaraj S. (2018). Gut microbiome in obesity, metabolic syndrome, and diabetes. Curr Diabetes Rep.

[72] Weyer C., Bogardus C., Pratley R.E. (1999). Metabolic factors contributing to increased resting metabolic rate and decreased insulin-induced thermogenesis during the development of type 2 diabetes. Diabetes.

[73] Alawad A.O., Merghani T.H., Ballal M.A. (2013). Resting metabolic rate in obese diabetic and obese non-diabetic subjects and its relation to glycaemic control. BMC Res Notes.

[74] St-Onge M.P., Gallagher D. (2010). Body composition changes with aging: the cause or the result of alterations in metabolic rate and macronutrient oxidation?. Nutrition.

[75] Suryadinata R.V., Wirjatmadi B., Adriani M., Lorensia A. (2020). Effect of age and weight on physical activity. J Public Health Res.

[76] Al-Sofiani M.E., Ganji S.S., Kalyani R.R. (2019). Body composition changes in diabetes and aging. J Diabet Complicat.

[77] Silver A.J., Guillen C.P., Kahl M.J., Morley J.E. (1993). Effect of aging on body fat. J Am Geriatr Soc.

[78] Zampino M., AlGhatrif M., Kuo P.L., Simonsick E.M., Ferrucci L. (2020). Longitudinal changes in resting metabolic rates with aging are accelerated by diseases. Nutrients.

[79] UPST Force (2021). Screening for prediabetes and type 2 diabetes: US preventive services task force recommendation statement. JAMA, J Am Med Assoc.

[80] Christensen P., Meinert Larsen T., Westerterp-Plantenga M., Macdonald I., Martinez J.A., Handjiev S. (2018). Men and women respond differently to rapid weight loss: metabolic outcomes of a multi-centre intervention study after a low-energy diet in 2500 overweight, individuals with pre-diabetes (PREVIEW). Diabetes Obes Metabol.

[81] Perreault L., Ma Y., Dagogo-Jack S., Horton E., Marrero D., Crandall J. (2008). Sex differences in diabetes risk and the effect of intensive lifestyle modification in the Diabetes Prevention Program. Diabetes Care.

[82] Heitzmann C.A., Kaplan R.M., Wilson D.K., Sandler J. (1987). Sex differences in weight loss among adults with type II diabetes mellitus. J Behav Med.

[83] Rentzeperi E., Pegiou S., Koufakis T., Grammatiki M., Kotsa K. (2022). Sex differences in response to treatment with glucagon-like peptide 1 receptor agonists: opportunities for a tailored approach to diabetes and obesity care. J Personalized Med.

[84] Risi R., Rossini G., Tozzi R., Pieralice S., Monte L., Masi D. (2022). Sex difference in the safety and efficacy of bariatric procedures: a systematic review and meta-analysis. Surgery for obesity and related diseases. Offc J Am Soc Bariatric Surg.

[85] Valaiyapathi B., Gower B., Ashraf A.P. (2020). Pathophysiology of type 2 diabetes in children and adolescents. Curr Diabetes Rev.

[86] Bays H., Scinta W. (2015). Adiposopathy and epigenetics: an introduction to obesity as a transgenerational disease. Curr Med Res Opin.

[87] Ling C., Ronn T. (2019). Epigenetics in human obesity and type 2 diabetes. Cell Metabol.

[88] Liu D., Huang Y., Huang C., Yang S., Wei X., Zhang P. (2022). Calorie restriction with or without time-restricted eating in weight loss. N Engl J Med.

[89] Fanti M., Mishra A., Longo V.D., Brandhorst S. (2021). Time-restricted eating, intermittent fasting, and fasting-mimicking diets in weight loss. Curr Obes Rep.

[90] Volaco A., Cavalcanti A.M., Filho R.P., Précoma D.B. (2018). Socioeconomic status: the missing link between obesity and diabetes mellitus?. Curr Diabetes Rev.

[91] Thornton P.L., Kumanyika S.K., Gregg E.W., Araneta M.R., Baskin M.L., Chin M.H. (2020). New research directions on disparities in obesity and type 2 diabetes. Ann N Y Acad Sci.

[92] Forhan M., Salas X.R. (2013). Inequities in healthcare: a review of bias and discrimination in obesity treatment. Can J Diabetes.

[93] Rubino F., Puhl R.M., Cummings D.E., Eckel R.H., Ryan D.H., Mechanick J.I. (2020). Joint international consensus statement for ending stigma of obesity. Nat Med.

